# Is there a Competition between Functional and Situational Affordances during Action Initiation with Everyday Tools?

**DOI:** 10.3389/fpsyg.2017.01073

**Published:** 2017-06-28

**Authors:** Kévin Roche, Hanna Chainay

**Affiliations:** ^1^Département Marketing, Grenoble Ecole de ManagementGrenoble, France; ^2^Laboratoire EMC, Université Lumière Lyon 2Lyon, France

**Keywords:** visuomotor priming, tool use, tool function, atypical tool use, affordance

## Abstract

Most studies of human-tool interactions focus on the typical use of a tool (e.g., cutting in the case of a knife). However, little is known about situations requiring atypical tool use (e.g., using a knife to tighten a screw). The present study focused on a selection of atypical uses of everyday tools which might be in conflict with their typical use. Our objective was to study how tool function influences the selection of the relevant action. In Experiment 1, which involved visuomotor priming, two everyday tools (a knife and a screwdriver) and two neutral tools (two bars, with no strong functional affordance) were used as primes and targets. Participants had to use the target with the appropriate box (indicated by the color) that allowed to make an action. Longer initiation times were observed when the prime was an everyday tool, irrespective of the nature of the target. We therefore observed a conflict between functional and situational affordances. To investigate whether the priming effect is caused by the task-irrelevance of the prime, we asked the participants in Experiment 2 to perform an action associated with the prime. The results showed longer initiation times only when the prime and target were everyday tools, irrespective of their precise nature. This suggests that activation of the typical use of a tool might not be fully automatic but flexible depending on the situation.

## Introduction

Throughout our lives, we interact with many tools in the same way to achieve the same goals (e.g., cutting food with a knife). These experiences allow us, among other things, to construct semantic/functional knowledge about tools (e.g., Buxbaum and Saffran, 1998; Borghi and Riggio, 2015). However, do we always need this functional knowledge to act with tools?

According to Gibson’s (1979) theory, it is the affordances, described as opportunities for action that are directly offered by the intrinsic perceptual properties of objects, that allow us to use tools. Since these properties are invariant, the affordances do not change as a function of our needs and goals. They are directly perceived without any need to call on previous experiences with the tool and knowledge of its function. This view of affordances was modified by authors who pointed out that they are relations between one’s abilities and features of the environmental situation (Tucker and Ellis, 2001; Chemero, 2003) and that they depend on previous experience and the current goal (Rosenbaum et al., 1990). Given this view, the perception of affordances depends on one’s needs in the specific situation at hand as well as on the ultimate aim of the action. For example, the affordance of a knife lies in the ability to cut food at lunchtime even though it may also be used to retighten the screw of one’s spectacles if needed.

Over the centuries, we have created many kinds of objects that recur consistently in our environment and lead to regular, routine actions related to their typical functions. It is very likely that this has led to us predominantly and automatically perceiving the recurrent functions of such objects. We use the term “functional affordances” to refer to this functional perception below. If we return to the example of a knife, we clearly use this object more often to cut food than to repair our glasses. However, functional affordances seem useless when a situation demands the atypical use of an object. In this case, it may be more appropriate to perceive situational affordances, which respond to the requirements of a situation and a goal that we wish to achieve. The purpose of the present study is to investigate whether functional affordances are automatically activated along with situational affordances when an atypical use of the tool is required.

Various evidence from behavioral (Derbyshire et al., 2006; McNair and Harris, 2012; Ni et al., 2014) and neuroimaging studies (e.g., Chao and Martin, 2000; Vingerhoets, 2008) lends support to the idea that humans directly and automatically perceive functional affordances in the presence of a common tool, whatever their intentions are. Visual processing of a tool is thought to be sufficient to activate the tool’s affordances in a bottom-up way (Tucker and Ellis, 1998, 2001; Yoon et al., 2002; Buxbaum and Kalénine, 2010; Jax and Buxbaum, 2010; Vainio et al., 2014; Kalénine et al., 2016) independently of one’s intention and the situation in which the action is performed.

Some interesting data about situation and goal-dependent affordance activation come from Jax and Buxbaum’s (2010, 2013) studies with tools (e.g., a calculator) evoking two competing affordances: structural (important for grasp-to-move gestures) and functional (important for grasp-to-use gestures). Jax and Buxbaum (2010) found longer initiation times (ITs) for the conflictual objects than for the non-conflictual objects, suggesting that both affordances are activated and that one of them has to be selected, thus slowing down action initiation. In addition, patients with ideomotor apraxia have been shown to find it more difficult to grasp conflictual tools than non-conflictual ones (Jax and Buxbaum, 2013). A recent study by Kalénine et al. (2016) investigated this conflictual effect in more detail by manipulating the distance between the observer and the tools. When the conflictual tools were presented out of the reachable space, the conflict between structural and functional affordances ceased to occur. These results suggest that functional affordances might be activated independently of the task and are not dependent on the situation (Lee et al., 2013).

Because our environment is constantly changing, we often need to adapt to the specific situation and its constraints. It is therefore possible that rather than always activating the typical use of the tools, people analyze the situation and how the tool may serve their purpose. The ability to perceive the situational affordance of the tool seems particularly important for planning actions and achieving one’s goal (Mizelle and Wheaton, 2010). It supports the flexibility of the human mind to achieve goals using the available resources and permits adaptation to new or unpredictable situations. In this perspective, it is the situation and the goal, not the typical function of the tool, that optimize tool use and processing (Phillips and Ward, 2002; Mizelle and Wheaton, 2010; Osiurak et al., 2010). In line with this view, it has been suggested that affordances are not automatically activated but are dependent on top-down processing determined by one’s motivation and goals in any given situation (Chemero, 2003, 2013; Costantini et al., 2010; Nonaka, 2013; Osiurak and Badets, 2014) as well as by the end state of the movement (Marteniuk et al., 1987; Rosenbaum and Jorgensen, 1992; see Rosenbaum et al., 2006 for a review). This selective modulation of affordance activation by the purpose of the action may help avoid the disruptive effects of competition between functional and situational affordances (e.g., Prinz, 1997; Cisek, 2007; Hommel, 2009; Cisek and Kalaska, 2010). For example, the study by Lindemann et al. (2006) suggests that functional affordances are only activated when subjects intend to grasp the tool in a functional way, as opposed to making a finger-lifting movement. Ranganathan et al. (2011) found that ITs were shorter when subjects were asked to grasp an upright glass in the normal way or with a magnetic implement than when the glass was upside-down. This effect was not present when participants touched the glass with their fist. The authors interpreted these results as evidence that tools do not activate functional affordances automatically but instead do so in the light of the situation and the intentions of the person performing the action.

In the present study, we investigated the activation of functional and situational affordances when atypical tool use was needed. To this end, and inspired by Jax and Buxbaum’s (2010, 2013) studies using tools presenting conflictual structural and functional affordances related to two different action goals (grasp-to-move and grasp-to-use) and involving different manipulations, we designed experimental material that made it possible to activate conflictual functional and situational affordances without involving different manipulations. We consequently used four stimuli: two common tools with a strong functional affordance (a knife and a screwdriver) and two control tools without any specific functional affordance (two wooden bars), together with two boxes designed to produce gestures similar to cutting and screwing, but not replicating the typical purpose of these actions. In this way, we were able, first, to control the grasp and the manipulation aspects of actions and make them as much as similar as possible for one and the same tool across different conditions, and, second, to create atypical, situational affordances. For each of the four tools, we created a new, situational affordance by associating it with a specific box having one of two colors (one common and one control tool were painted in red and were associated with a red box and the equivalent was done for a blue box). The common tools had two potentially conflicting affordances (one functional and one situational), while the control tools only had a situational affordance. In Experiment 1, we used a visuomotor priming paradigm in which the common and control tools were presented as primes and targets in order to reveal any prejudicial effect of prime processing on action ITs for the target. The participants’ task was to use the target with the appropriate box, i.e., blue target with the blue box and red target with the red box. The target color therefore indicated the goal of the action. The pairs of common tools in the prime and target formed the conflictual condition in which the functional affordance (activated by the common tool in the prime) could conflict with the situational affordance required by the task. Our hypothesis was that if functional affordances are automatically activated by the presence of a common tool, then they should conflict with situational affordances and consequently slow down the selection of the situational affordance. We therefore expected to observe longer ITs for conditions with a common tool in the prime and/or target than in conditions in which the same control tool (bar) was present in the prime and target. On the other hand, if functional affordances are not activated automatically and situational affordances are activated by the prime, we should observe faster ITs when both prime and target have the same color, independently of their identity.

## Experiment 1

### Method

#### Participants

Twenty students (16 women) from Lyon 2 University took part in the present study. Their mean age was 21.5 years (*SD* = 2.9). Participants were divided into two equal groups according to the category of tool (common tool versus control tool) used as target. All of them were self-reported as right-handed, with normal or corrected-to-normal vision. With regard to ethics, members of the laboratory gave their approval for the experiments presented in this study. In addition, prior to taking part in the experiment, the participants gave their written, informed consent in accordance with the Helsinki declaration.

#### Material and Stimuli

**FIGURE 1:**
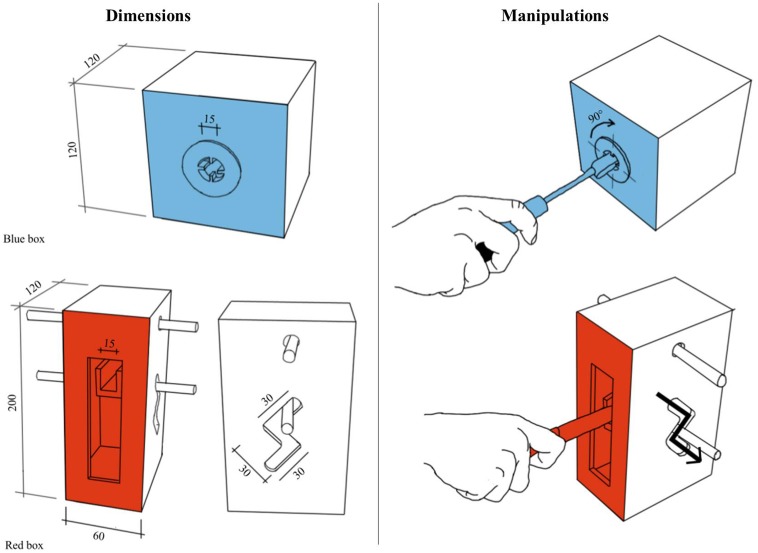
Dimensions and manipulation associated with the two experimental boxes.

A Dell computer equipped with E-prime2^TM^ software (Psychology Software Tools, Inc., United States) was used to run the experiment and record the movement IT. The liquid-crystal goggles (Plato Translucent Technologies, Toronto, ON, Canada) used to control the subjects’ vision were connected to the computer, along with a home-made, 4-cm diameter spherical release button which was used to collect the ITs. The tools used as prime and target were placed on a board 40 cm wide × 50 cm long. Two boxes were designed in order to produce gestures similar to cutting with a knife and screwing with a screwdriver (**Figure 1**). The boxes were placed at the left of the board, at a distance and angle that made it easy for the participants to interact with them. The “screwing” box was a black cube with a blue front. A piece of plastic was inserted in the middle of the front in such a way that it could be rotated in both directions. The “cutting” box was a black cube with a red front. In the middle of the front was a “Z”-shaped slit. Inside the box, there was a small horizontal platform (held in place with elastic bands) that could be reached through the upper part of the slit and moved downward along a “Z”-shaped track.

The common tools (knife and screwdriver) and control tools (bars) presented as prime and target were painted to match the colors of the boxes. The screwdriver and one bar were blue while the knife and the other bar were red. To avoid acoustic cues about the nature of the prime and target, small pieces of felt were attached to the ends of tools in contact with the experimental board.

#### Procedure

The participants were tested individually. They were positioned to face the experimental board, with their right hand on the release button. The primes and targets were presented on the experimental board one at a time, at a distance of about 40 cm from the participant and in front of their right shoulder. The handles of the tools were turned toward the participants and at 45° to the right of their midline. Before the experiment started, the participants were asked to get used to using the tools with the two boxes for as long as they judged necessary in order to perform the actions quickly and efficiently. The participants were divided into two groups depending on the Category of Tool used as target: common tools vs. control tools. Consequently, one group of participants saw common tools as targets and the other group saw control tools as targets. The two groups were formed in such a way that it would be easy to dissociate between possible target effects and priming effects. Each participant performed 10 training trials followed by 64 experimental trials. There were eight experimental conditions (four prime conditions × two target conditions), each of which was repeated eight times. The order of the trials was pseudorandomized across participants.

The instruction was to use the target with the appropriate box as a function of the target and box color (a blue target with a blue box, a red target with a red box). More specifically, when working with the blue box, the participants were asked to turn the middle piece 45° to the right, whereas with the red box, they were told to push the small platform down along the “Z” track. All the trials started with a “beep” to remind the participants to place their hand on the release button. At the same time, the goggles became opaque for 1500 ms, during which period a prime was placed on the experimental board. The goggles then became transparent for 500 ms so that the prime was visible, before becoming opaque again for a further 1500 ms. During the ISI, the experimenter removed the prime from the experimental board and replaced it with the target. At the end of the ISI, the goggles became transparent again and a simultaneous “go” signal indicated to the participants that they were to grasp the target as quickly as possible and use it in the corresponding box before putting it back on the board. The participants were given 7000 ms to perform the task. When the prime and the target were the same, the experimenter always displaced the prime so that the participants were not able to predict that the upcoming target was the same tool as a prime.

There were two repeated-measures factors: Target (Blue-Box-Compatible: BBC versus Red-Box-Compatible: RBC) and Prime (Common-Tool BBC – screwdriver; Common-Tool RBC - knife; Control-Tool BBC – blue bar; Control-Tool RBC – red bar); and one between-subject factor Target-Tool-Category (Common-Tool versus Control-Tool). Concerning the Target factor, in the BBC condition the target was either a common or control tool to be used with the blue box (screwing action), while in the RBC condition, the target was either a common or control tool to be used with the red box (cutting action).

We measured IT as the time that elapsed between the “go” signal and the time when the participants took their hand off the release button. Preliminary analyses conducted to check for normality (Shapiro–Wilk’s test) and sphericity (Mauchley’s test) detected no violations. ITs above 1000 ms, below 150 ms, or differing by more than 2.5 standard deviations from the individual mean for each condition were removed (less than 2% of the data). A mixed-measure ANOVA was performed with one between-subject factor: Target-Tool-Category and two within-subject factors: Target and Prime. A significance level of *a* = 0.05 was used for all statistical analyses. The participants performed the task accurately, with an overall accuracy rate of 96.5%. For control purposes, we checked for a possible difference between the two boxes, but found no significant difference.

### Results

**FIGURE 2:**
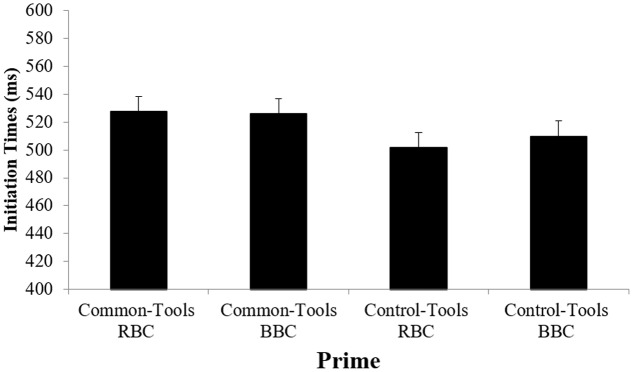
Mean Initiation Times (ITs) as a function of Prime condition: common tool RBC, common tool BBC, control tool RBC and control tool BBC. Error bars represent 95% within-subject confidence intervals.

The ANOVA showed a significant effect of the Prime [*F*(3,54) = 3.74, *p* < 0.02, η^2^ = 0.17; **Figure 2**]. Planned comparisons revealed significantly longer ITs after the presentation of common tools than control tools in the prime. More precisely, the IT was longer after common RBC primes than after control RBC primes (*p* < 0.04) and marginally longer than after control BBC primes (*p* = 0.07). Similarly, the IT were significantly longer after common BBC primes than after control BBC primes (*p* < 0.05) and control RBC primes (*p* < 0.04). There was no other significant difference.

The other effects and interactions were not significant. More specifically, the simple effects of Target [*F*(1,18) = 2.13, *p* = 0.16] and Target-Tool-Category [*F*(1,18) = 0.22, *p* = 0.64] and the Prime^∗^Target [*F*(3,54) = 0.84, *p* = 0.47], Target^∗^Target-Tool-Category [*F*(1,18) = 2.12, *p* = 0.16], Prime^∗^Target-Tool-Category [*F*(3,54) = 1.71, *p* = 0.17] and Prime^∗^Target^∗^Target-Tool-Category [*F*(3,54) = 0.29, *p* = 0.82] interactions were not significant.

### Discussion

In Experiment 1, we formulated two alternative hypotheses. One was that if the activation of affordances depends on the requirements of the situation and the individual’s goals, then functional affordances should not be activated automatically. Instead, only situational affordances should be activated. Consequently, shorter ITs should be observed when prime and target share the same color. Our results do not support this hypothesis as we did not observe significantly shorter ITs in these conditions. Indeed, the interaction between prime and target was not significant, indicating that action ITs were not faster when the prime and target were compatible (e.g., red bar in prime and target, knife in prime and target).

The second hypothesis was that functional affordances are activated automatically independently of the situation and the participants’ goals. This hypothesis led to the prediction of longer ITs for conditions with a common tool as prime because common tools should activate both functional and situational affordances, and functional affordances should therefore compete with situational affordances, thus slowing down action ITs. In accordance with this prediction, the results showed longer ITs when common rather than control tools were presented as primes, irrespective of the target category (common or control tool) and prime-target compatibility. This might suggest that seeing a common tool in the prime automatically activates its functional affordance and influences the processing of the target, even if the function of the tool is irrelevant to the situation. Our data are consistent with Lee et al.’s (2013) and Kalénine et al.’s (2016) observations suggesting that functional affordances may be activated automatically.

It might be expected that if functional affordances are activated automatically then seeing a common tool as a target should also have some prejudicial effects on the time of action initiation because, in this case, functional affordances might compete with situational demands. However, our data do not indicate such disruptive effects given that we did not observe any significant effect of Target-Tool-Category or any significant interaction of this factor with Prime. The ITs were not significantly longer when the target was a common tool as compared to a control tool in any of the prime conditions. In particular, our results indicate that the conflict between functional and situational affordances was not greater for a condition in which the tool was the same in the prime and target. It is possible that functional affordances were automatically and predominantly activated when common tools were presented as the prime because the participants did not have to perform an action in response to the prime and processing of the prime, which was irrelevant to the task, was not influenced by the participants’ goals and intentions. However, when we consider the tools presented as the target, which were relevant for the task because the participants were asked to use them, there was no automatic activation, because it was counteracted by the situation-dependent processing of task-relevant information. Thus, when the situation requires an atypical, situational use of a tool, the functional affordance is probably not activated sufficiently to interfere with the more highly activated required situational affordance. On the other hand, it could be argued that if situational affordances are activated only when they are task-relevant, it is possible that they are not activated during the processing of the prime because, as we have already said, the participants did not have to perform any action with the prime. To test this idea, it would be possible to ask the participants to perform an action using the target as if it were the tool seen in a prime. Thus, in Experiment 2, we used a protocol similar to that used in Experiment 1 and we asked the participants to use the target in the same way as if it were the prime they had just seen. Our prediction was that if the activation of functional affordances is counteracted by that of situational affordances then the ITs should be shorter for common tools when the prime and target share the same color. This should also be the case for control tools because they only activate situational affordances.

We also formulated an alternative prediction. According to the view that the dominance of functional affordances means that they should be automatically activated whatever the situation, longer action ITs should be observed when common tools are presented as primes, and especially when both prime and target are common tools. In this case, functional affordances would enter into competition with situational affordances.

## Experiment 2

### Method

#### Participants

Twenty students (15 women) from Lyon 2 University took part in the present study. Their mean age was 22.6 years (*SD* = 2.7). All of them were self-reported as right-handed, with normal or corrected-to-normal vision. Prior to taking part in the experiment, the participants gave their written, informed consent in accordance with the Helsinki declaration. The participants were divided equally into two groups depending on the nature of the prime (common and control tools).

#### Material and Stimuli

The same material and stimuli were used as in Experiment 1.

#### Procedure

The procedure was similar to that used in Experiment 1. However, each group of participants was presented with only one type of prime (common or control tool). In this experiment, the participants were instructed to use the target. However, the way in which they used it was determined by the prime. Thus, for example, if the prime was a common tool to be used with the blue box (screwdriver), then the target had to be used as a screwdriver with the appropriate box (blue box) irrespective of whatever it actually was (knife, screwdriver, red or blue bar). The four tools were presented as targets.

A mixed-measures ANOVA was performed, with a between-subject factor: Prime-Category (Common Tool vs. Control Tool) and two within-subject factors: Prime (Blue-Box-Compatible: BBC vs. Red-Box-Compatible: RBC) and Target (Common-Tool BBC – screwdriver; Common-Tool RBC - knife; Control-Tool BBC – blue bar; Control-Tool RBC – red bar).

The participants performed the task accurately, with an overall accuracy rate of 94.2%. For control purposes, we checked for a possible difference between the two boxes, but found no significant difference.

### Results

**FIGURE 3:**
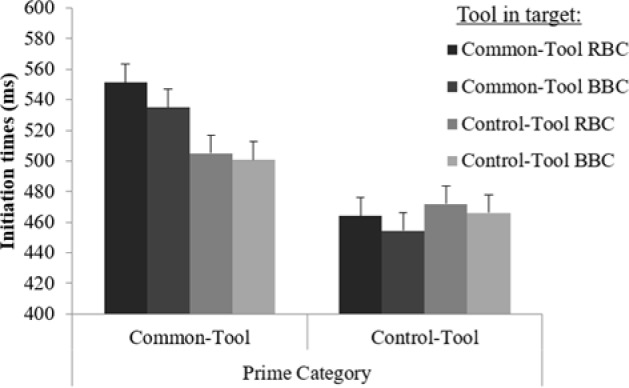
Mean ITs in Experiment 2, as a function of prime category (common tool, control tool) and target (common tool RBC, control tool RBC, common tool BBC, control tool BBC). Error bars represent 95% within-subject confidence intervals.

The ANOVA showed no significant simple effects and no significant interactions (all *p* > 0.1), except for the interaction between Prime Category and Target [*F*(3,54) = 4.36, *p* < 0.01, η^2^ = 0.19; **Figure 3**]. Planned comparisons showed that when the primes were common tools, ITs were significantly longer for common tool than for control tool targets. More precisely, longer ITs were observed for common RBC targets (mean = 551 ms) than for control RBC targets (mean = 504 ms; *p* < 0.04) or for control BBC targets (mean = 500 ms; *p* < 0.05). Similarly, common BBC targets (mean = 534 ms) had longer ITs than control BBC (*p* < 0.02) and control RBC cargets (*p* < 0.01). There was no significant difference between common BBC targets and common RBC targets (*p* = 0.23) or between control BBC targets and control RBC targets (*p* = 0.62). No significant differences were observed in the action ITs when control tools were presented as primes between the different target conditions.

In addition, we looked at differences between the prime categories (common vs. control tools) for each target condition. Comparisons revealed marginal differences for both common tools (RBC: *p* = 0.07; BBC: *p* = 0.08) and no significant difference for control tools (RBC: *p* = 0.44; BBC: *p* = 0.44).

### Discussion

In Experiment 2, we formulated two alternative hypotheses. The first posited that situational affordances would be activated in response to a prime only if this information is relevant for the performance of an action with a target and that this activation would counteract that of functional affordances. We therefore asked our participants to use the target in the same way as if it were the prime and we expected to observe shorter ITs in conditions in which the same common or control tool was presented in the prime and target than in conditions in which different tools were presented. Our results did not support this prediction given that no facilitating effect of compatibility between prime and target was observed on action initiation. Although, the ITs were in general slightly faster when control rather than common tools were used in the prime conditions, the effect of the Prime was not significant.

The second hypothesis was that functional affordances are activated automatically, independently of the situational demand and that they will conflict with situational affordances. We therefore expected to observe longer action ITs in a condition in which a common tool is presented as the prime, and especially when both prime and target are common tools. Our results confirmed our prediction. In fact, longer action ITs were observed when a common tool was presented as the prime, and this finding increased when the target was also a common tool.

Given that in the present experiment the prime indicated the action goal that had to be maintained in working memory until target presentation, there is reason to wonder whether our results might have been influenced by limitations to working memory capacity (Baddeley, 1992, 2003; Vandierendonck, 2016). However, if the processing of irrelevant information is dependent on the resources available in working memory, asking participants to perform an action in the light of the viewed prime should increase the memory load and consequently leave fewer resources available for the processing of functional affordances (Heuer et al., 2016). Thus, the fact that only a low level of resources was available in working memory might have decreased the activation of irrelevant affordances and, consequently, have caused common tool primes to have a less disruptive effect on action initiation (Randerath et al., 2013; Vainio et al., 2014; Grgic et al., 2016). However, our data do not seem to be in agreement with this hypothesis.

## General Discussion

Everyday tools are specific in having a typical function. For example, a glass is typically used to drink from. Sometimes, however, depending on the situation, a tool may be used in an atypical fashion. For instance, we can use a glass to catch a wasp before it becomes a nuisance or, worse still, stings us. In this case, we regard the glass as a trap with the opening at the bottom and no longer as a drinking vessel. The purpose of the present study was to gain a better understanding of the way tools are processed in a context of atypical use, and more precisely during the processing of situational and functional affordances.

In two experiments, participants had to use common tools, which had typical functional affordances, or control tools, which had no such affordances, in combination with boxes involving a new goal (i.e., situational affordances). The way the tools were used with the boxes differed from the typical use of the tools in terms of the end-goal of the action, but not in terms of the grasping and movement gestures. Our main hypothesis was that the conflict between functional and situational affordances should occur if the tool function is automatically activated and that this should be expressed through longer ITs in conditions in which the tool is presented in the prime or target. In general, our results in both experiments confirmed this hypothesis. They therefore suggest that the typical function of a tool may be activated automatically and may consequently disrupt the selection of the relevant action.

It is well known that a tool can activate different affordances (Bub et al., 2008) and that to execute the required action, it is necessary to select the appropriate affordance (Cisek, 2007; Pezzulo and Cisek, 2016). While some studies have focused on conflicts created by the differences in affordances linked not only to the goal but also to the performed gestures – for example the grasp-to-move and grasp-to-use gestures for a calculator are different (Jax and Buxbaum, 2010, 2013; Borghi et al., 2012; Kalénine et al., 2014) –, the present study investigated conflicts created by the differences linked only to the goals, one of which was typical (related to tool function) and the other situational (related to situational demand) and both involving similar gestures. Our results indicate that different gestures are not necessary in order to induce a conflict and that it is sufficient to have different goals. This is consistent with the view that holds that intentions and goals influence visuomotor processes at different levels, from the most abstract plan to more precise parameters (Chemero, 2003; Cisek and Kalaska, 2005; Nonaka, 2013).

To better discuss the extent to which tool function is necessary for action selection in specific situations and the ways in which it may conflict with situational demands, it seems important to specify what the tool function is. Sensorimotor theories suggest that the specific function of a tool is a part of our knowledge about the tool (Buxbaum, 2001; Gallese, 2005; Barsalou, 2008). Such function knowledge is activated automatically irrespective of the situation (Kalénine et al., 2016) and makes it easier to select the typical goal amongst alternative (or atypical) goals (Buxbaum, 2001). Action initiation is simplified if a situational use of a tool is consistent with its typical function. In other cases, the effects of this type of automatic activation are more likely to be disruptive. The effects observed in our study are consistent with the sensorimotor approach. In fact, we observed longer action ITs when common tools were presented as the prime, thus suggesting that functional affordances were activated automatically and somehow entered into conflict with the situational demand. However, and surprisingly, this prejudicial effect on action initiation was not observed when the tool was presented as the target, the processing of which was therefore relevant for the task. It is somewhat difficult to explain these results within the visuomotor framework. The ideomotor approach, which proposes that tool function knowledge is better explained in terms of a framework involving a relationship between the tool and the goal in specific situations (i.e., between a knife and a loaf of bread or between a screwdriver and a screw) (Mizelle and Wheaton, 2010; Osiurak et al., 2010; Baber et al., 2014), seems more appropriate. For Baber et al. (2014), tool use is guided by the goal in response to a specific need and the consequences of this use. It can therefore be suggested that if an action has to be performed using a tool then the situational affordances are predominantly activated and inhibit functional affordances.

Another explanation of our results may be that the activation of functional affordances is related to the gesture that is to be performed rather than to the action goal. In our experiments, functional and situational affordances led to different goals, while the way the tools were grasped and manipulated were very similar. However, the idea that the function of a tool is more closely related to the gesture than to the goal seems somewhat incompatible with studies suggesting that knowledge of tool function can be learned without performing any gestures and instead simply through visualization of the action and its consequences (Jeannerod and Jacob, 2005). This kind of learning is supported by the mirror neurons, which are specific in their ability to process not only sensory and motor information but also the goal of the action (Kohler et al., 2002; Gallese, 2005). The goal of the action can therefore be processed directly and learned without the gesture. It therefore seems that knowledge about the function of a tool is derived from the goal rather than from the gesture (von Hofsten, 2007).

However, it is possible that planning an action activates the processing of relevant motor information, even if the information comes from a stimulus other than the action target (Lindemann et al., 2006). This suggestion is consistent with the view predicting that relevant motor information processing depends on one’s intentions and plans (Allport, 1987). Thus, the intention to act in a precise situation could, at a very early stage of information processing, activate a general sensitivity to certain motor components (Massen and Prinz, 2009; van Elk et al., 2010; Przybylski and Króliczak, 2017). In the case of our study, in which grasp and movement were very similar for both functional and situational affordances, activation during processing of the task-relevant motor components in response to the prime might have entered into competition with automatically activated task-irrelevant functional affordances. As we have explained above, the activation of functional affordances when a tool is present in the prime would not be counteracted by the activation of situational affordances, because these would be activated only when an action is required and this was not the case for the primes in Experiment 1. The data from Experiment 2, in which the goal of the action was determined by the prime (the participants were asked to act with the target as if it were the prime they had just seen) are compatible with this explanation, given that longer action ITs were observed in the condition in which a common tool was presented as the prime and were even longer when the target was also a common tool.

To summarize, our study contributes new information to the discussion about the automaticity of the activation of functional affordances. We suggest that this activation might not be fully automatic but might be flexible depending on the situation (Cisek, 2007; Borghi et al., 2012; Borghi and Riggio, 2015; Pezzulo and Cisek, 2016). More precisely, to avoid conflicts between the processing of different types of affordances, the activation of functional affordances may depend on the extent to which a tool is relevant for a task. In this way, no conflict emerges for one and the same tool and the processing of relevant information when an action is initiated may be thought of as adaptive and economical.

## Author Contributions

All authors listed, have made substantial, direct and intellectual contribution to the work, and approved it for publication.

## Conflict of Interest Statement

The authors declare that the research was conducted in the absence of any commercial or financial relationships that could be construed as a potential conflict of interest.
